# Patient and Surgeon Satisfaction Levels after Using an Acrylic, Hydrophobic, Monofocal IOL and the Malyugin Ring in Pseudoexfoliation Syndrome Patients

**DOI:** 10.1155/2018/3843098

**Published:** 2018-05-15

**Authors:** Andreas F. Borkenstein, Eva-Maria Borkenstein

**Affiliations:** Private Practice, Privatklinik der Kreuzschwestern Graz, Kreuzgasse 35, 8010-Graz, Austria

## Abstract

**Purpose:**

The purpose of this prospective analysis was to evaluate the patient and surgeon satisfaction levels after using a fully preloaded acrylic, hydrophobic, monofocal “premium” IOL in pseudoexfoliation syndrome.

**Materials and Methods:**

42 eyes of twenty-eight patients with progressed cataract formation and pseudoexfoliation syndrome (PXF) or pseudoexfoliation glaucoma and best corrected distance visual acuity (BCDVA) between 0.30–1.00 logMAR were enrolled. After a detailed preexamination, they underwent phacoemulsification and implantation of the acrylic, hydrophobic, heparin-coated, single-piece, monofocal intraocular lens (CT LUCIA 601P, Zeiss, Germany) with 360° square edge and ultraclear purity aspheric ZO optic. We evaluated the visual performance of the IOL and the patient satisfaction. We also evaluated the intraoperative handling of the injector and the behavior of the IOL in these complicated cases (PXF).

**Results:**

The BCDVA increased from mean 0.48 logMAR (range 0.30–1.00 logMAR) preoperatively to −0.05 ± 0.13 logMAR postoperatively. The mean IOL power was 23.5 D (range 16.5–27.5 D). The target refraction using the Haigis formula within ±0.5 D was reached by 92.9% (*n*=39) and by 100% (*n*=42) within 1.0 D of all cases, respectively. Patient satisfaction was very high, and no halos or glare were reported in any case. The fully preloaded injector system enabled an easy IOL preparation and safe implantation.

**Conclusion:**

Our results show that the implantation of the fully preloaded CT LUCIA 601P (Zeiss, Germany) is safe and enhances OR workflow in complicated cases as pseudoexfoliation. In these cases, an adapted approach (special preoperative, intraoperative, and postoperative regime) with considering possible complications is necessary to achieve best outcomes.

## 1. Introduction

**Figure 1:**
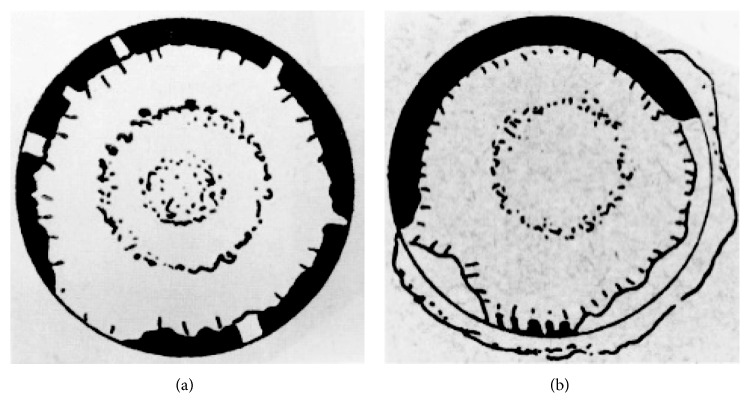
Hand drawings of Lindberg: The central disc and peripheral band are illustrated as well as the degeneration of the pupillary border.

**Figure 2:**
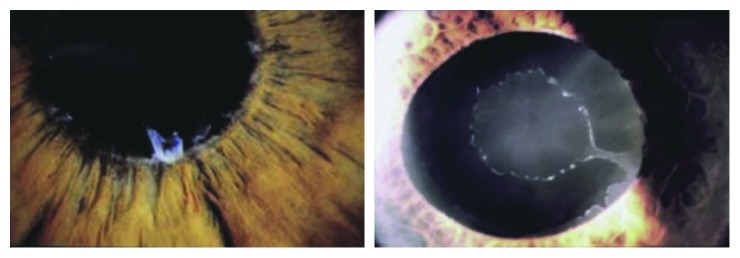
Pseudoexfoliation syndrome with grey-whitish flakes and PXF disc.

The pseudoexfoliation syndrome (PXF) was first described in 1917 by the Finnish ophthalmology resident Lindberg. Lindberg modified a Zeiss microscope and constructed a slit lamp on his own because slit lamps were not commercially available at that time. He conducted a study of more than 200 patients and recorded his findings with skilful hand drawings (Figures 1 and 2), and his conclusions still stand valid today [1]. Unfortunately, Lindberg was not cited in subsequent research projects by his colleagues as he was a modest gentleman who did not complain [2].

The prevalence of PXF increases with age; it is seldom seen before the age of 50. Pseudoexfoliation syndrome has been reported from all over the world, although prevalence varies between countries and even between local geographical areas. Prevalence rates may depend on the diagnostic criteria used and the age group of the population studied [3]. PXF syndrome and PXF glaucoma, which must be promptly detected to avoid surgical complications, are frequent in patients scheduled for cataract surgery. Regarding a study of consecutive cases, in 25.2% of patients, exfoliation was detected in the preoperative examination [4].

Cataract surgery in eyes with pseudoexfoliation syndrome is associated with higher rates of complications. Therefore, accurate preoperative precautions and exact intraoperative care are required to ensure a successful and safe surgery with the best postoperative outcomes [5, 6].

## 2. Materials and Methods

**Figure 3:**
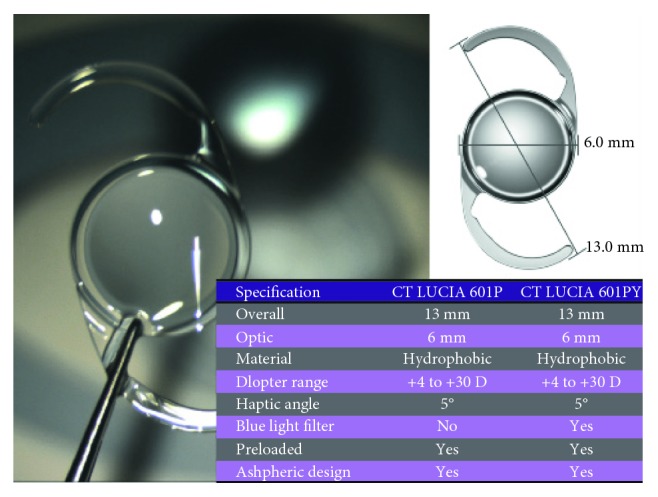
Specifications of the acrylic, hydrophobic CT LUCIA 601(P) (Zeiss, Germany).

In this prospective analysis, 42 eyes with progressed cataract formation (senile cataract) and a best corrected distance visual acuity (BCDVA) between 0.30 and 1.00 logMAR were enrolled. The main inclusion criteria was an existing and detectable pseudoexfoliation syndrome with its characteristic findings in the anterior segment which includes the typical PXF disc with its fluffy deposits on the anterior lens capsule, the transillumination defect on the pupillary borders, and the poor pupillary dilation (maximum pupil diameter 3.5 mm in spite of medical-induced mydriasis). The intraoperative application of the 6.25 mm Malyugin ring to enlarge the pupil was mandatory, which is another inclusion criterion. 27 eyes were diagnosed with pseudoexfoliation glaucoma, high intraocular pressure (IOP) regulated with topical glaucoma medication, and pathognomonic glaucoma-associated defects in optic nerve disc with visual field loss. The cloudy lenses were graded with the “LOCS III” (Lens Opacities Classification System) in nuclear, cortical, and subcapsular opacities. Exclusion criteria included other severe eye pathologies such as corneal dystrophy, posttraumatic corneal wound scar, diabetic proliferative retinopathy, age-related macular degeneration, or retinal dystrophy. In all cases, phacoemulsification and removing of the cloudy lens were performed with the same phacomachine (Abbott Signature Whitestar) by the same surgeon in the same facility. Repeated measurements were performed preoperatively to avoid or minimize complications of cataract extraction and get the best outcomes in these PXF eyes. An increased awareness was required because of the problem this disorder may induce. Biometry was performed with the IOLMaster 500 (Zeiss, Germany) twice to increase accuracy even in hard nuclear or dense subcapsular cataracts. In 4 cases, ultrasound measurement of the axial length (Axis) was necessary. In all cases, Scheimpflug measurement with the Pentacam AXL (Oculus, Germany) for exact evaluation of the corneal topography, keratometry, and pachymetry was performed. Objective measurements of the refractive error including autorefraction and IOP measurements with Goldmann tonometry were performed at least 3 times (1–12 weeks) before surgery. Perimetry results (visual field) were meaningful and utilizable in just 8 cases, and in all the other cases, the dense cataract had already blocked too much light and the visual acuity was worse. A special preoperative procedure of eye drops was applied: 3 days prior to surgery, patients received bromfenac (NSAID) eye drops (2 times daily) and cyclopentolate eye drops 3 hours prior to surgery. Tetracaine eye drops (4x) as topical anesthesia were instilled 30 minutes before surgery, and disinfecting eye drops were used several times. In all cases, the fully preloaded, acrylic, hydrophobic, heparin-coated, single-piece, monofocal, “premium IOL”-CT LUCIA 601P (Zeiss, Germany) was implanted without any complications (Figure 3). The lens had an overall diameter of 13 mm (C-loop with 5° angulation) and a 360° square edge design with clear, aspheric ZO optic. Additionally, in all cases, the 6.25 mm “Malyugin ring” was inserted through the main incision and placed to provide a large and round pupil. Control examinations including visual tests for BCDVA, slit lamp exam, IOP measurement, and Scheimpflug measurement for effective lens position were performed after 1, 4, 7, 28, and 84 days. In addition, imaging (photograph documentation) of the IOL and optical coherence tomography (OCT) of the macula were done one week and three months after surgery. In all cases, the postoperative medication included acetazolamide 250 mg tablets 1 × 1 for the first 2 days, bromfenac eye drops (2 weeks), and betamethasone sodium phosphate eye drops for 4 weeks.

## 3. Results

**Figure 4:**
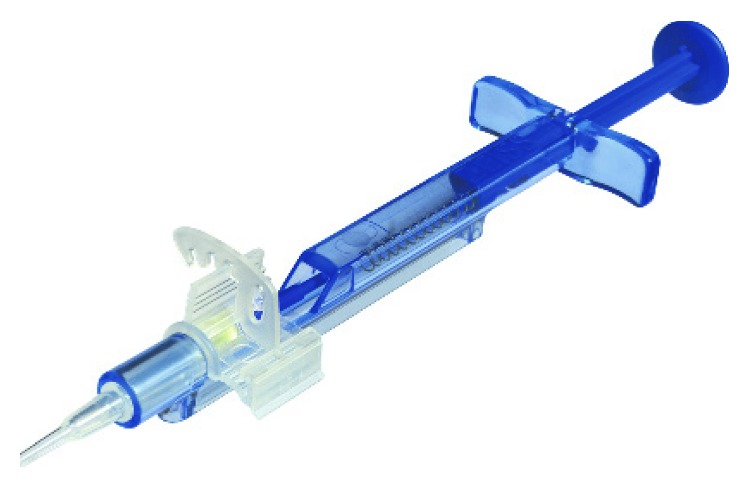
Fully preloaded injector.

42 eyes of twenty-eight patients (19 female, 9 male) with dense cataracts and pseudoexfoliation syndrome were included. The mean age of the participants was 77.9 years ± 7.5 years. The cloudy lenses (cataracta senilis) were graded with the “LOCS III” (Lens Opacities Classification System) in 16 nuclear (8x NC5 and 8x NC6), 14 cortical (3x C3, 5x C4, and 6x C5), and 12 subcapsular posterior (5x P4 and 7x P5) lens opacities. The best corrected distance visual acuity (BCDVA) increased from mean 0.48 logMAR (range 0.30–1.00 logMAR) preoperatively to −0.05 ± 0.13 logMAR postoperatively. The mean IOL power was 23.5 D (range 16.5–27.5 D). The target refraction with Haigis formula was reached within ± 0.5 D by 92.9% (*n*=39) and within 1.0 D by 100% (*n*=42) of all cases, respectively. Patient satisfaction was very high, and no halos or glare were reported in any of the cases. The fully preloaded injector system (Zeiss, Germany) enabled an easy and safe IOL preparation and good OR workflow (Figure 4). The implantation of the CT LUCIA 601P was performed “wound-assisted” (just the tip of the nozzle goes in the corneal tunnel) through a 2.4 mm clear cornea incision. The insertion of the acrylic, hydrophobic, single-piece IOL was achieved, despite the small pupil and Malyugin ring without touching the iris or the ring directly into the capsular bag without further manipulation. The IOL-unfolding process was slow, controlled, analogue, and independent of the IOL power. The unfolding of the trailing haptic did not interfere with the Malyugin ring, and no “handshake phenomenon” (sticking of the haptics to the optic) was recognized. The desired lens orientation and positioning was achieved fast with very minimal capsular and zonular stress. No complications (*n*=0) in terms of posterior capsule defects or (sub)luxation of the lens were experienced. Intraocular pressure (IOP) was independent from medications in pseudoexfoliation syndrome patients, and pseudoexfoliation glaucoma patients showed lower values after lens extraction with a mean decrease of 4 mmHg 3 months postoperatively. These findings showed a significant correlation to the anterior chamber depth measured with the Pentacam (mean 2.95 mm preoperative versus 4.08 mm postoperative values). The lens position image analysis of retroillumination photos of 40 eyes showed well-centered IOLs, and the effective lens position (ELP) did not change significantly over the postoperative course of 3 months. In two cases, an intense capsular phimosis (8 weeks after surgery) with “myopic shift effect” led to a slight displacement of the IOL in the direction of the anterior chamber with resulting myopia (−1.0 D). The slit lamp exam and retroillumination photo showed a clear acrylic optic with high purity without any glistenings.

## 4. Discussion

Pseudoexfoliation syndrome is a systemic, age-related disease and occurs worldwide, but the prevalence varies between different regions. PXF is a risk factor for both glaucoma (≈30%) and cataract formation. Moreover, several studies have verified that PXF is correlated with an increased risk of intraoperative and/or postoperative complications [7]. The deposition of extracellular fibers results in alterations of tissues in the anterior segment of the eye which makes cataract surgery potentially challenging [8]. Surgeons must be aware of numerous preoperative, intraoperative, and postoperative issues in managing the PXF eyes. For extensive surgical considerations in matters of the pathophysiology of PXF, the changes and their impact should include the following:

1. A smaller pupil with posterior synechiae causes reduced visualization and complicates the procedure. NSAIDs can be given preoperatively to discourage intraoperative miosis. The application of the Malyugin ring through the main incision (2.2 mm) is a very elegant and time-saving method to achieve a round, large pupil without damaging the iris sphincter and without the need of making extra paracentesis. Especially in the PXF eyes, the surgical procedure should be as gentle as possible. To enlarge the pupil and to separate the synechiae, cohesive viscoelastic devices (OVDs) can be used. The use of trypan blue for staining the anterior capsule can be very wise in cases of poor red-reflex in dense subcapsular cataracts.
2. Zonular weakness and phacodonesis can make the capsulorhexis, phacoemulsification,irrigation/aspiration, and implantation of the IOL challenging. Depending on the severity of the zonular dialysis, a capsular tension ring (CTR) should be used in the intraoperative course as soon as possible to facilitate the situation.
3. Less anterior capsule tension makes the capsulorhexis in the PXF eyes more difficult. The filling of the anterior chamber with OVD should be repeated, and the rhexis should be done with a sharp needle or forceps very slowly and carefully to generate as less pressure to the zonules as possible. The size and design of the rhexis is most important for good IOL centration because of potential postoperative shrinkage and phimosis in the PXF eyes.
4. Phacoemulsification has to be performed with special respect for the weak zonules and the thinner posterior lens capsule. A chopping technique with low ultrasound energy and low irrigation/aspiration adjustments is recommended. The cortical removal is a crucial step in the PXF eyes. The “Hurricane I/A technique” allows a tangential vector resultant voltage on the zonules smaller than the conventional radial suction. In some cases, the “dry” removal of cortex with a cannula (stripping technique) is the best way to preserve zonular fibers.
5. Several studies have confirmed that the acrylic, hydrophobic IOLs are the best choice in pseudoexfoliation syndrome. Considerations should include the diameter of the lens, the haptic design and IOL stability, the edges regarding the PCO rate, and the surface of the lens. There are studies with controversial results regarding the effect of a heparin coat, and its benefit has not been confirmed till now [9]. Further investigations have to be done. However, we are certain that the clarity and purity of the optic is most important. Any inclusions in the lens material such as glistenings can also lead to light scatter effects, halos, and glare. In the PXF eyes, the possibility of IOL exchange is limited because of the higher risk of surgical trauma to the tissues [10]. When we are afraid of the higher likelihood of postoperative decentration and tilt of the IOL (caused by phimosis or pseudophacodonesis), we should not select negative aspheric aberrated IOLs because if the optic position is not well aligned, other higher order aberrations (HOAs), such as coma, could occur and reduce contrast sensitivity and effect quality of vision.
6. The implantation of the IOL should be as harmless as possible with a minimum of stress to the zonules, less manipulation in the anterior chamber, and fast orientation/centration of the IOL in the capsular bag [11]. Therefore, the right injector is also important. The ideal insertion of the IOL should be as harmless as possible regarding the weak zonules and altered capsular bag. Sometimes, it is advantageous to form a kind of “viscoshield” (combination of cohesive and dispersive OVD) in the capsular bag just before implantation. Hence, the posterior capsule is protected from the unfolding process of the leading haptic which is slower and more controllable for the surgeon. Any additional manipulation with instruments (spatula, Sinsky hooks, and so on) should be avoided. Moreover, especially in complicated cases, a good OR workflow can improve the atmosphere in the operating room and the mood of the surgeon and the scrub nurse subsequently. Best results can be achieved with careful and slow approach.
7. It is well known that, in pseudoexfoliation, the intraocular pressure may rise up rapidly 2 hours after surgery. Persistent intraocular pressure elevations and persistent inflammation in the first postoperative period are very common [12]. Therefore, a special adapted postoperative regime is recommended. Rebound tonometry without topical anesthesia, which is fast, simple, safe, and reliable can be done either in the sitting or lying position. Intraocular pressure should be measured before discharge and the first morning after surgery.
8. With the rising life expectancy, the zonules are finally lost from the epithelium and the bag with the IOL may loose into the vitreous. It was found that 6-7 years following cataract surgery, posterior chamber intraocular lenses were positioned lower in pseudoexfoliation syndrome than in control eyes [13]. Due to possible (sub)luxation of the lens, photodocumented slit lamp examinations should be performed carefully and more frequently. In this way, a prompt intervention can be initiated.

## 5. Conclusion

Pseudoexfoliation syndrome is a common disorder, and every cataract surgeon has to face the special challenges associated with it. Careful preoperative considerations and special intraoperative care to ensure safe surgery are necessary to get the best postoperative outcomes.

Our prospective analysis in cataract patients with pseudoexfoliation syndrome and dense cataracts showed that the implantation of the fully preloaded CT LUCIA 601P (Zeiss, Germany) is safe and enhances OR workflow in complicated cases. Postoperatively, no halos or glare were reported by the patients. We have not observed a single case of glistenings till now, and therefore, we fully recommend the CT LUCIA 601P in complicated cases, and we believe that the label of “premium” is appropriate.

## Additional Points

*Data Availability.* The data used to support the findings of this study are included within the article.
